# Systolic heart failure and cardiac resynchronization therapy: a focus on diastole

**DOI:** 10.1007/s10554-014-0412-1

**Published:** 2014-04-05

**Authors:** Emanuela Facchini, Marco Varalda, Chiara Sartori, Daniel Burkhoff, Paolo Nicola Marino

**Affiliations:** 1 Clinical Cardiology, Department of Translational Medicine, Azienda Ospedaliero Universitaria “Maggiore della Carità”, Università del Piemonte Orientale, Corso Mazzini 18, 28100 Novara, Italy; 2 Division of Cardiology, Columbia University, 177 Fort Washington, Avenue, New York, NY 10032 USA

**Keywords:** Cardiac resynchronization therapy, Heart failure, Diastolic function

## Abstract

Conflicting data exist about the effects of cardiac resynchronization therapy (CRT) on diastolic function (DF). Aim of the study was to assess if and how CRT affects DF in systolic heart failure patients. We also investigated potential relations between CRT-induced left ventricular changes and the composite clinical endpoint of progressive heart failure and cardiac death over 3 years follow-up. 119 CRT patients underwent clinical evaluation and echocardiography before CRT and 4 months later. DF was quantified by transmitral velocities [E/A waves, deceleration time (DT), E/DT], early diastolic mitral annulus velocity (E′), E/E′ ratio and 2-D speckle tracking strain rate during isovolumetric relaxation (IVR, SRivr). End-diastolic pressure–volume relationship (EDPVR) was also assessed noninvasively using a single-beat method. Overall stiffness was quantified by ventricular stiffness (K_lv_) normalized to end-diastolic volume (EDV). New York Heart Association class improved at 4 months (from 2.7 ± 0.7 to 1.9 ± 0.6, *p* < 0.001) as did ventricular filling (E/DT from 0.48 ± 0.29 to 0.39 ± 0.31 cm/s^2^, *p* = 0.01). In contrast, relaxation (E′, SRivr) and filling pressures (E/E′, E/SRivr) did not change. Slope of EDPVR did not change with CRT. Such finding, together with an unmodified Klv/EDV and a 7 ± 18 % reduction in EDV (*p* = 0.001), suggested reverse remodelling towards a smaller equilibrium volume. Finally, end-systolic LV volume decreased from 147 ± 59 to 125 ± 52 ml and ejection fraction increased from 0.26 ± 0.07 to 0.32 ± 0.09 (both *p* < 0.001). Using a Cox regression model we found that only changes (Δ) in diastolic, but not systolic indexes, correlated with the composite clinical endpoint, with increments in ΔEDV20 and ΔE/DT, single or combined, greatly increasing risk of heart failure and/or cardiac death (*p* = 0.003). Ventricular reverse remodelling, together with improvement in ventricular filling, rather than improvements of systolic function, predict clinical prognosis long-term post-CRT.

## Introduction

Cardiac resynchronization therapy (CRT) improves functional capacity, left ventricular systolic function and survival in patients with chronic heart failure and left bundle branch block [1]. Not much, however, is known about the effects of CRT on diastolic function (DF) in these patients. This is surprising given that the increase in left ventricular filling pressure highly correlates with the degree of exercise limitation in patients with chronic heart failure, independent of the severity of systolic dysfunction [2]. Furthermore, the few previous studies of the impact of CRT on DF have presented variable and contrasting results [3–5].

Thus, the aim of the present study was to assess if and how CRT impacted DF in systolic heart failure patients. DF was assessed using echocardiographic *load*-*dependent* as well as relatively *load*-*independent* parameters. In addition, we analyzed the passive phase of ventricular diastole by the end-diastolic pressure–volume relationship (EDPVR) using a single-beat approach that uses noninvasively-estimated diastolic pressure and volume data [6, 7]. Since CRT can induce significant ventricular reverse remodelling [8], a non load-dependent index of EDPVR such as operative ventricular stiffness (K_lv_) [9] normalized to end-diastolic volume (EDV) was also used. Finally, we investigated potential relations between CRT-induced changes in diastolic parameters and clinical recurrence of heart failure and/or cardiac death over a follow-up of 3 years.

## Methods

### Population

One hundred eight five (185) patients with systolic heart failure (ejection fraction ≤35 %) and a clinical indication for CRT were retrospectively identified from our clinic. In all patients, informed consent had been previously obtained in accordance with institutional human studies committee guidelines. From this initial group we excluded patients with biological or mechanical mitral valves, atrial fibrillation or high heart rates that precluded a clear separation of E versus A waves on mitral inflow velocity acquisitions. No statistical differences in clinical and echocardiographic parameters between included and excluded patients were observed, apart from the proportion of AICDs versus pacemakers, which was higher in the included patients (*p* = 0.001) (Table 1).

**Table 1:** Population’s clinical characteristics

	Included patients	Excluded patients	*p* value
Patients (n)	119	66	
Age (years)	70 ± 9	71 ± 8	0.24
Gender (n, %)	Male 96 (81 %)	Male 49 (74 %)	0.41
	Female 23 (19 %)	Female 17 (26 %)	
Etiology (n, %)	Ischemic 63 (53 %)	Ischemic 27 (41 %)	0.16
	Nonischemic 56 (47 %)	Nonischemic 39 (59 %)	
NYHA class	2.7 ± 0.7	2.8 ± 0.7	0.41
Quality of life score	27.3 ± 21.5	32,4 ± 23,4	0.49
Six minute walking test (m)	350 ± 114	302 ± 87	0.12
QRS (ms)	158 ± 25	164 ± 28	0.11
EDV (ml)	195 ± 66	179 ± 59	0.09
ESV (ml)	147 ± 59	134 ± 52	0.10
EF	0.26 ± 0.07	0.26 ± 0.07	0.46
CRT-device (n, %)	AICD 109 (92 %)	AICD 48 (73 %)	0.001
	PaceMaker 10 (8 %)	PaceMaker 18 (27 %)	

*AICD* automatic internal cardiac defibrillator, *CRT* cardiac resynchronization therapy, *EDV* end-diastolic volume, *EF* ejection fraction, *ESV* end-systolic volume, *NYHA* New York Heart Association

Thus, our final population (Table 2) included 119 patients (mean age 69.8 ± 8.9 years, 81 % males) suffering from heart failure due to various aetiologies, who were candidates for CRT according to latest ESC guidelines (ejection fraction ≤35 %, QRS ≥120 ms and NYHA functional class II–III despite optimized medical therapy) [1]. Ischemic cardiomyopathy was defined as a documented previous myocardial infarction or significant coronary artery disease (luminal narrowing >50 %) at coronary arteriography. Optimal revascularization had been performed in these patients. Nonischemic aetiology was defined only in the presence of angiographically normal coronary arteries or a negative stress-rest thallium scan.

**Table 2:** Population’s baseline heart rate, aetiology, comorbidity and therapy

Heart rate (beats/min)	70 ± 14
Aetiology of heart disease (n)	
Hypertensive	1
Ischemic	63
Valvular	8
No obvious cause	47
Therapy (n)	
ACE-inhibitors/AT1 antagonists	97
Amiodarone	29
Antialdosterons	38
Anticoagulants	13
Antiplatelets	78
Beta-blockers	88
Ca^++^ channel blockers	16
Digitalis	18
Diuretics	83
Nitrates	40
Proton pump inhibitors	63
Statins	55
Comorbidity (n)	
Hypertension	68
Diabetes	39
Vasculopathy	35
COPD	25
Renal failure	27
Liver disease	9

Vasculopathy: previous ischemic ictus attack and/or previous carotid artery thromboendarterectomy and/or previous aortic aneurysmectomy and/or AOCP (chronic obstructive peripheral arteriopathies)

*NYHA* New York Heart Association, *COPD* Chronic Obstructive Pulmonary Disease

Patients were subjected to a transthoracic echocardiographic examination using standard equipment (Vivid 7 or Vivid E9, GE Medical System, Horten, Norway). Cardiac cycles were stored in digital format and then subjected to an off-line analysis using dedicated software (EchoPAC PC version BT10, GE Healthcare). Patients were imaged before implantation (29 ± 46 days) and after 4 months (125 ± 73 days). We evaluated left ventricular end-systolic and end-diastolic volumes and ejection fraction. In addition, we acquired extensive evaluation of diastolic echocardiographic parameters to quantify ventricular relaxation, filling pressures and left ventricular stiffness by continuous-Doppler, pulsed and tissue Doppler according to published guidelines [10]. Furthermore, we quantified ventricular dyssynchrony using Temporal Uniformity of Strain (TUS) Index applied to longitudinal strains from 2D speckle-tracking echocardiographic images. [11].

Patients also underwent a clinical evaluation at baseline and after 4 months to assess NYHA functional class, to complete a Minnesota Living with Heart Failure Questionnaire (MLWHFQ) and perform a 6 min walk test. During subsequent long-term clinical follow-up (980 ± 667 days) exacerbations of heart failure causing hospitalization or activation of “OPTIVOL”, available in 39 patients, requiring additional diuretic administration or death were recorded.

### Echocardiographic measurements

Ventricular volumes were calculated in biplane mode using Simpson’s method applied to images obtained in the apical 4- and 2-chamber views. The degree of mitral regurgitation was assessed calculating the area of the regurgitant jet on colour-Doppler images (4- and 2-chambers) and expressed as a percentage of the left atrium area.

**Fig. 1:**
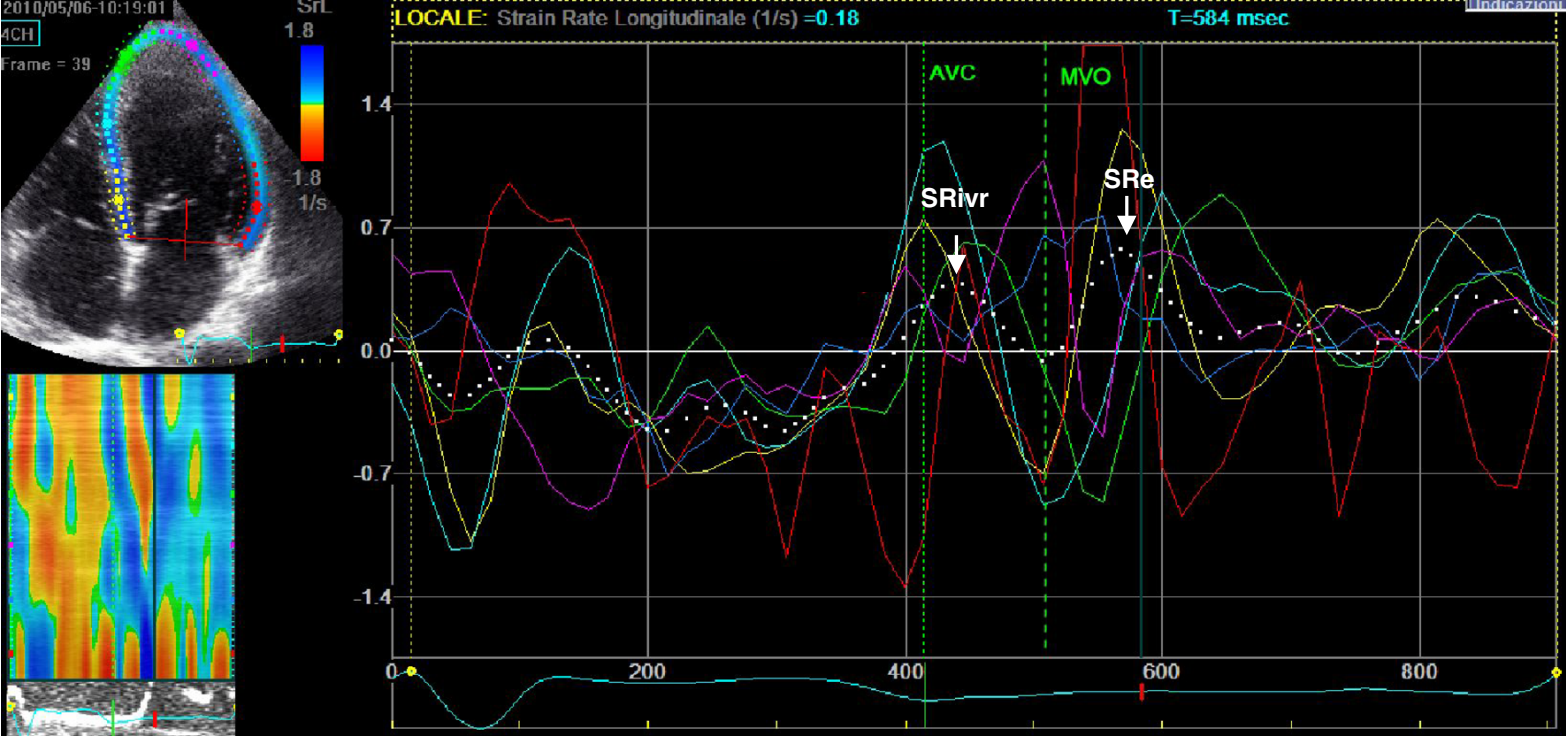
Strain rate analysis using spleckle-tracking echocardiography applied to a 4-chamber view in a patient. The mean derivative curve of strain (*white*, *dotted line*) shows two diastolic peaks. The *first peak* represents the global peak strain rate during isovolumetric relaxation (SR_ivr_), while the *second peak* represents strain rate during early ventricular filling (SR_e_). *AVC* aortic valve closure valve closure, *MVO* mitral valve opening

Diastolic function was characterized by classical *load*-*dependent* and relatively *load*-*independent* parameters. Classical *load*-*dependent* parameters included: transmitral early and late diastolic velocity (E and A waves), E-wave deceleration time (DT), E/A and E/DT ratio assessed using pulsed Doppler in 4-chamber view. Relatively *load*-*independent* parameters included: early diastolic mitral annulus velocity (average of septal-lateral-front and posterior annulus E′) obtained using pulsed tissue Doppler in the apical 2- and 4-chamber views. E′ can be considered as a ventricular relaxation index [10], while the E/E′ ratio represents a noninvasive estimation of ventricular filling pressures [12]. Colour M-mode Doppler mitral flow propagation velocity (Vp) was also obtained in a 4-chamber projection [13]. We were able to calculate this parameter in 53 patients only. We also assessed longitudinal strain rates during isovolumetric relaxation (IVR) and in the early phase of diastolic filling [14]. Such parameters were obtained applying a speckle-tracking algorithm to 4- and 2-chambers apical projections, with the endocardium manually traced and software automatically drawing epicardial edges in order to identify the region of interest and to derive a strain curve. The first derivative of the strain curve presents two diastolic peaks: the first peak (after aortic valve closure) represents global peak strain rate during isovolumetric relaxation (SR_ivr_) and is an index of relaxation rate [15] (Fig. 1). The second peak represents strain rate during early ventricular filling (SR_e_) and it is affected by the final balance between ventricular relaxation and atrial pressure [14]. Finally we identified the IVR interval by taking into account the aortic and mitral valve openings and closings with pulsed wave Doppler. IVR interval was reported on the strain graph in order to identify SR_ivr_. E/SR_ivr_ ratio was also computed and taken as a measure of ventricular filling pressures.

Ventricular dyssynchrony was indexed pre and 4 months post CRT by TUS, whereby a time plot of regional strains, arranged for ventricular location, is subjected to a Fourier analysis. If segments shorten simultaneously (synchronously), the plot appears as a straight line, with power only in the zero-order Fourier term, whereas regionally clustered dyssynchrony generates an undulating plot with higher power in the first-order term. TUS index reflects zero-order relative to first-order plus zero-order power [11]. We and others have shown that baseline asynchrony and coincidence between the latest mechanical activation site and left ventricular lead position predict favourable prognosis after CRT [11, 16, 17].

Finally we assessed ventricular *passive properties* according to a single-beat end-diastolic elastance algorithm which allows noninvasive estimation of the EDPVR from EDV and end-diastolic pressure (EDP) [6, 7]. According to this algorithm the EDPVR of any healthy or sick heart can be estimated by a nonlinear analytical expression: EDP = α.EDV^^^β where EDP is obtained from E/E′ according to the following equation [EDP = 1.91 + (1.24·E/E′)] [12]. Values obtained in this manner were then normalized to fixed values of EDP (10, 20, 30 mm Hg) in order to compare subjects and make comparisons; [EDV_10_, EDV_20_, EDV_30_ = (EDP/α)^^1/β^, with EDP = 10, 20, or 30 mmHg, respectively].

It is recognized, indeed, that there are limitations in E/E′-based measurements of filling pressures in patients with conduction abnormalities or undergoing pacing [18]. Thus, passive ventricular properties were also quantified by K_lv_, which was determined from DT according to the following equation (K_lv_ = 1.28^exp[−0.008DT]^); this approach has been validated by invasive methods in a group of comparable cardiomyopathic patients [9]. Values were then normalized to EDV (K_lv_/EDV).

### Device implantation

All patients received a biventricular pacemaker (10 CRT-P, 109 CRT-D) with lead positions in conventional locations (right atrium, right ventricle and the coronary sinus). Implanted devices were: 18 GUIDANT (Renewal^®^), 38 MEDTRONIC (12 Concerto HF^®^, 17 Consulta CRT^®^, 5 Insync^®^, 4 Sentry^®^), 38 SAINT JUDE (20 Atlas HF^®^, 11 Promote Accel^®^, 2 Epic HF^®^, 2 Frontier 2^®^, 2 Unify^®^, 1 Anthem^®^), 9 BIOTRONIK (Lumax HF^®^), 6 SORIN-ELA (4 Paradyn CRT^®^, 1 Newliving CHF^®^, 1 Ovatio CRT^®^) 10 BOSTON SCIENTIFIC (Cognis^®^). All subjects underwent echocardiographic-guided atrioventricular optimization 2–3 days after implantation (mean atrioventricular interval 133 ± 30 ms). Interventricular delay was set fixed at 0 ms.

### Statistical analysis

Data are expressed as mean ± standard deviation. Differences between means were assessed using *t* tests for paired data. Signed rank tests were used if data were not normally distributed. Chi square test was used to compare proportions. Univariate and multivariate Cox regression analyses were used to evaluate the relationship between echocardiographic parameters with significant variations pre- and post-CRT (with the exception of age, gender, aetiology of cardiac disease and QRS duration) versus recurrence of heart failure or death (whichever came first) during a 3 year follow-up period. Survival analysis was performed by Kaplan–Meier survival analysis with significance testing using log-rank statistics and a post hoc Holm–Sidak test. A two-way repeated-measures ANOVA was finally used to assess the effects of CRT on time changes in Vp, with the attribution to responder versus nonresponder group as a between-patient factor. A *p* value <0.05 was considered as statistically significant. Analyses were performed using Sigmaplot (version 12.5 for Windows, Jandel; San Rafael, CA) statistical software.

### Variability of analysis

Inter-observer variability of echo-Doppler parameters was determined from 30 randomly selected patients and assessed as absolute mean difference ± the percentage coefficient of variation (SD/mean). Reproducibility was: 6.6 mL ± 0.93 % for EDV, 0.04 cm/s^2^ ± 1.1 % for E/DT, 0.92 ± 0.75 % for E/E′, 0.02 s ± 1.2 % for IVR, 0.06 s^−1^ ± 9.4 % for SR_ivrt_, 0.09 s^−1^ ± 1.55 % for SR_e_ and 0.15 ± 1.2 % for TUS longitudinal index.

## Results

All clinical parameters improved significantly after CRT (NYHA from 2.7 ± 0.7 to 1.9 ± 0.6, quality of life score from 27.3 ± 21.5 to 17.6 ± 19.9, 6 min walking test from 350 ± 114 min to 393 ± 107 min, *p* < 0.001 for all). QRS and heart rate decreased (−4.4 ± 20.8 and −2.4 ± 19.4 %, *p* = 0.005 and *p* = 0.01, respectively). At 4 months (Table 3) echocardiographic derived findings improved as well, showing decreases in ventricular volumes, better ejection fraction and reduced mitral regurgitation. TUS index, measured in the longitudinal axis, increased after CRT (+12 ± 40 %, *p* = 0.035, Table 3), suggesting amelioration of baseline dyssynchrony.

**Table 3:** Echocardiographic parameters before and 4 months after CRT

	Basal	4 months	*p* value
End-systolic volume (ml)	147 ± 59	125 ± 52	<0.001
End-diastolic volume (ml)	195 ± 66	178 ± 59	<0.001
Ejection fraction	0.26 ± 0.07	0.32 ± 0.09	<0.001
MI area/left atrium area	0.17 ± 0.15	0.12 ± 0.13	<0.001
Dyssyncrony (TUS index)	0.59 ± 0.15	0.62 ± 0.15	0.035
E wave (cm/s)	75 ± 30	65 ± 26	<0.001
A wave (cm/s)	69 ± 27	70 ± 23	0.85
E/A	1.41 ± 1.16	1.15 ± 0.93	0.045
DT (ms)	190 ± 78	217 ± 90	0.038
E/DT (cm/s^2^)	0.48 ± 0.29	0.39 ± 0.31	0.01
IVR (s)	0.125 ± 0.05	0.132 ± 0.05	0.014
E′ (cm/s)	5.29 ± 1.81	5.39 ± 1.84	0.90
SR_ivr_ (s^−1^)	0.09 ± 0.15	0.11 ± 0.16	0.44
E/E′	16.7 ± 10.5	14.8 ± 10.7	0.23
SR e (s^−1^)	0.369 ± 0.176	0.361 ± 0.171	0.37
E/SR_ivr_	548 ± 2,895	165 ± 1,837	0.55
EDV_10_ (ml)	173.9 ± 62.6	163.3 ± 61.7	0.043
EDV_20_ (ml)	194.2 ± 70.0	181.2 ± 65.0	0.017
EDV_30_ (ml)	207.5 ± 75.5	192.9 ± 68.6	0.013
α Coefficent EDPVR	2.7E−0.8 ± 3E−0.7	6E−0.9 ± 4E−0.8	0.10
β Coefficent EDPVR	8.4 ± 13.9	5.7 ± 5.3	0.33
Klv/EDV [(mmHg/ml)/ml]	0.0017 ± 0.0009	0.0016 ± 0.0010	0.47

*CRT* cardiac resynchronization therapy, *DT* deceleration time, *EDPVR* end-diastolic pressure–volume curve, *EDV*_*10*–*20*–*30*_ end-diastolic ventricular volume at 10–20–30 filling pressures, *IVR* isovolumetric relaxation time, *K*_*lv*_ operative ventricular stiffness, *MI* mitral insufficiency, *SR*_*e*_ strain rate E, *SR*_*ivr*_ isovolumetric strain rate

*Load*-*dependent* left ventricular filling parameters improved too: E wave velocity decreased (−9.8 ± 28.6 cm/s, *p* < 0.001), as did the E/A ratio (−0.4 ± 1.2, *p* = 0.045). Due to an increment in DT, the E/DT ratio decreased significantly (−0.09 ± 0.33 cm/s^2^, *p* = 0.01) suggesting improved filling characteristics.

Noninvasive left ventricular filling pressure measurements tended to decrease, but insignificantly (E/E′ −1.4 ± 9.9 cm/s; SR_e_ +0.0026 ± 0.22 s^−1^; E/SR_ivr_ −592 ± 3,384; estimated EDP from 22.4 ± 12.6 mmHg to 20.1 ± 12.9 mmHg; ns for all). Furthermore, no significant changes could be detected for the diastolic relaxation indices E′ and SR_ivr_ (Table 3). IVR increased post-CRT (+0.014 ± 0.05 s, *p* = 0.014).

**Fig. 2:**
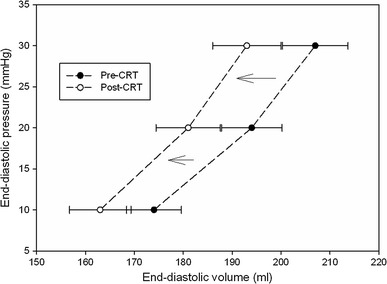
CRT-induced effects on ventricular diastolic volumes (mean ± SE) at comparable filling pressures (10–20–30 mmHg). Graph suggests a CRT-mediated leftward shifting towards a smaller equilibrium volume with no change in EDPVR slope, compatible with a reverse remodeling effect

As far as the diastolic passive properties were concerned (Fig. 2), at 4 months follow-up there were significant (*p* < 0.05) reductions in EDV_10_ EDV_20_ and EDV_30_, with no change in slope coefficients of EDPVR, consistent with an unmodified K_lv_/EDV. As shown in the figure and Table 3, there was a significant shift of the estimated EDPVR to smaller volumes, indicative of reverse remodeling.

### Clinical outcomes

Follow-up data, obtained from hospital records and/or telephone interviews with patients or their relatives, were available for all but 1 subject, who was lost at follow-up. Three more patients died for unknown reasons before any information about their clinical status prior to death could be acquired and were thus excluded from survival analysis There were 44 patients that reached the end-point (13 deaths, 26 patients with heart failure hospitalizations, 5 patients with “OPTIVOL” alarm activations followed by additional diuretic administration).

According to the univariate results (accepted level *p* < 0.1) of the Cox regression analysis ΔE/DT (*p* = 0.013), taken as a cumulative descriptor of ventricular filling, ΔEDV_20_ (*p* = 0.021), expression of the amount of CRT-induced ventricular reverse remodeling [19], and changes in end-systolic volume (ΔESV, *p* = 0.002), used as an index of ventricular performance, were included, as continuous covariates expressed as a percent change, in the multivariate analysis. Gender and etiology of ventricular dysfunction (ischemic/nonischemic) were also included as categorical covariates together with age and duration of QRS, gender acting as a stratum [20]. The analysis showed that progressive ventricular remodeling (ΔEDV_20_% *p* = 0.053) and worsening in left ventricular filling characteristics (ΔE/DT % *p* = 0.053) predicted heart failure exacerbations or death during the follow-up. Patients’ age (*p* = 0.015) and ischemic etiology (*p* = 0.013) contributed too. There was no significant contribution from ΔESV % or QRS duration (*p* = 0.609 and *p* = 0.682, respectively).

In order to confirm that ΔEDV_20_ and ΔE/DT (both measurements available in 84 patients) affected survival after CRT, we performed a Kaplan–Mayer analysis classifying the CRT population on a ΔEDV_20_ [−7.47 ml (−4.34 % relative to pre CRT)] plus an ΔE/DT [−0.05 cm/s^2^ (−18 % relative to pre CRT)] entire population median basis. Thus, 3 groups were created according to the values of ΔEDV_20_ and ΔE/DT compared to the related medians (group 1, n = 22: ΔEDV_20_ <5 % and ΔE/DT <18 %; group 2, n = 41: ΔEDV_20_ <5 % and ΔE/DT >18 % or ΔEDV_20_ >5 % and ΔE/DT <18 %; group 3, n = 21: ΔEDV_20_ >5 % and ΔE/DT >18 %).

**Fig. 3:**
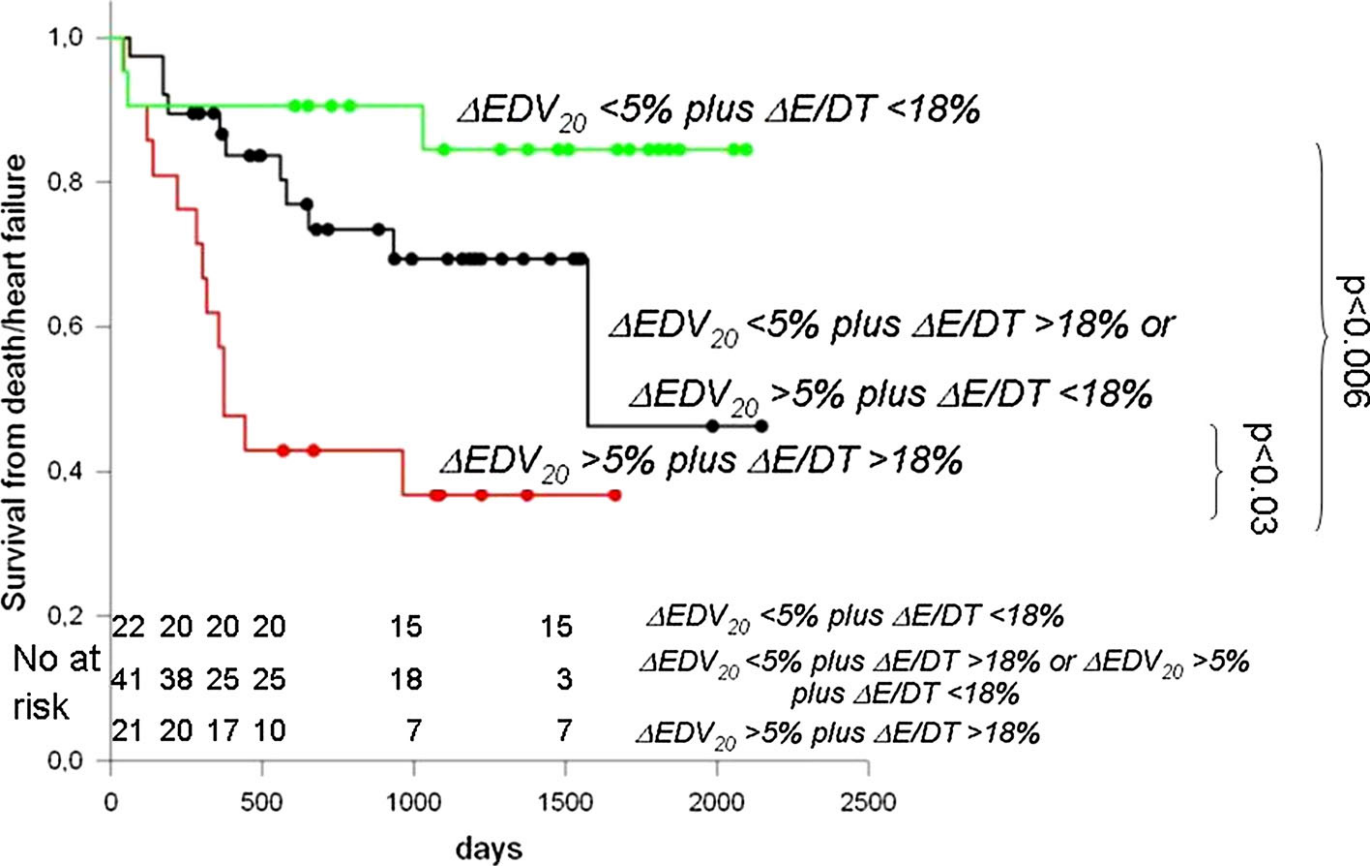
Survival curves obtained dividing patients into three groups according to values of ΔEDV_20_ and ΔE/DT compared to the related medians. Group 1: ΔEDV_20_ <5 % and ΔE/DT <18 %, group 2: ΔEDV_20_ <5 % and ΔE/DT >18 % or ΔEDV_20_ >5 % and ΔE/DT <18 %, group 3 ΔEDV_20_ >5 % and ΔE/DT >18 %. Group 3 survival is half of that of group 1 at the end of follow-up. Furthermore, group 3 survival rapidly decreases in the first 500 days after CRT. In group 1 instead, survival from heart failure and cardiac death is maintained around 90 % until the end of the observation. The difference is statistically significant (*p* < 0.006). Group 2 exhibits an intermediate trend, with an improved survival as compared with group 3 (*p* < 0.03) but worse than group 1, although not at a significant level (*p* = 0.113, ns)

Event-free survival curves for the three groups, shown in Fig. 3, were significantly different (log-rank test *p* = 0.001). Group 3 event-free survival was half of that of group 1 at the end of follow-up. Furthermore, group 3 event-free survival rapidly decreased in the first 500 days after CRT. In contrast, group 1 event-free survival was maintained around 90 % until the end of the observation period. The difference, based on a post hoc Holm–Sidak test was statistically significant (*p* < 0.006). Group 2 exhibited an intermediate trend, with improved event-free survival as compared with group 3 (*p* < 0.03), but worse relative to group 1, although not at a significant level (*p* = 0.113, ns). The results could not be explained by differences in the drug distribution among the three groups. The percentage of patients treated with beta-blockers (85, 75, 68 %), diuretics (76, 73, 77 %) and ACE inhibitors/AT1 antagonists (86, 85, 86 %) was not dissimilar among the 3 groups (*p* = 0.995).

## Discussion

Our study demonstrates that CRT does not significantly affect relaxation phase (E′ and SR_ivr_), nor filling pressures (E/E′ and E/SR_ivr_) in patients affected by dilated cardiomyopathy and systolic heart failure at 4-month follow-up. CRT, however, induces left ventricular reverse remodelling, resulting in a smaller ventricle with improved filling characteristics. Both dyssynchrony and systolic function improve after CRT, as demonstrated by TUS index and ejection fraction increments versus systolic volume decrements, but such changes do not seem to influence patients’ outcome long-term in a significant way.

Previous studies have demonstrated that CRT improves DF only in patients with an associated improvement in systolic function [3, 4]. However, data regarding CRT effects on diastole are controversial. Waggoner et al. [4] evaluated DF in a cohort of 50 heart failure patients receiving CRT using conventional echocardiographic measurements before and after 4-month follow-up. They defined as “responders” patients with an >5 % increase in ejection fraction at 4-month. Transmitral parameters (E, E/A, DT) and E/E′ ratio showed significant improvements only in responders. E′, an indirect index of relaxation, did not change in either group. The authors concluded that CRT exerts a beneficial effect on LV filling pressures but does not affect relaxation properties.

Jansen et al. [3] evaluated DF after up to 12 months of CRT. Both diastolic *load*-*dependent* and relatively *load*-*independent* measurements (respectively E, E/A, DT, E′ and E/E′) were significantly improved only in responders, defined as patients with a reduction >15 % in LV systolic volume at 12-month follow-up. In contrast to Waggoner et al., they concluded that reverse remodelling induced by CRT was associated with an improvement in both relaxation and filling pressures. Along the same line of thinking, Aksoy et al. [21] documented an improvement in filling pressures, estimated using the E/E′ ratio, only in responders defined as patients with a reduction ≥10 % in LV systolic volume at 6-month follow-up after CRT.

Porciani et al. [22] classified 65 patients according to the transmitral profile in restrictive and non restrictive filling patterns (25 vs. 40 patients). At 12-month follow-up after CRT implantation E/A ratio increased and DT decreased in the non-restrictive filling pattern group. In the restrictive group, instead, 13 patients showed a reduction in E/A and an increase in DT, while in the remaining patients the restrictive filling pattern did not vary. All cause mortality rate was 5 % in non restrictive filling pattern group, 15 % in patients with reversible restrictive filling pattern and 42 % in patients with persistent restrictive filling profile. These authors concluded that CRT improves DF in a considerable number of patients with filling restriction at implantation, but persistence of the restriction pattern after CRT is associated with worse outcome.

Shanks et al. [15] evaluated DF using speckle-tracking imaging, in addition to conventional methods. One hundred eighty-eight heart failure patients were evaluated before CRT implantation and at 6-month follow-up. Responders were defined as patients with a decrease in LV systolic volume ≥15 % 6 months after implantation. Among conventional echocardiographic parameters only DT showed an improvement in both responders and non responders. All the remaining parameters, such as E, E/A, E′ and E/E′, did not show any significant changes. SR_ivr_ and E/SR_ivr_ ratios, obtained with speckle-tracking analysis, improved only in responders, suggesting that CRT has beneficial effects on both relaxation and filling pressures.

Retracing Shanks’ study, our aim was to evaluate DF in patients with dilated cardiomyopathy and to verify if and how improvements after CRT, if any, affect patients’ outcomes. In line with previous studies, we documented an improvement in transmitral flow parameters, which are *load*-*dependent*. In contrast with data in literature, however, neither relaxation parameters (E′ and SR_ivr_) nor indirect filling pressure descriptors (E/E′ and E/SR_ivr_) showed statistically significant improvements. Only IVR increased significantly.

As mentioned before, ventricular diastolic relaxation is influenced by both myocardial inactivation and asynchrony [23]. Since asynchrony decreased after CRT, as documented by the improvements in TUS index, it is difficult to substantiate why this improvement does not affect relaxation parameters. A first interpretation may be that although CRT reduces asynchrony, it does not modify intracellular factors (such as SERCA quantity) involved in the process of pressure decay during IVR, in contrast with other experimental studies [24]. This conclusion is consistent with prior animal studies showing no change in the rate of relaxation despite large changes in the degree of ventricular synchrony [25]. In a more realistic scenario the modifications induced by CRT are minor and not easily inferable from noninvasive indices, with limited reliability in quantification of ventricular relaxation.

### Effects of CRT on ventricular passive properties

In contrast to previous noninvasive studies, we evaluated ventricular passive properties and how they were affected by CRT. After having computed EDV_10_, EDV_20_ EDV_30_ and K_lv_/EDV we can conclude that CRT induces reverse remodelling with no change in slope of EDPVR (Table 3). Such shift towards a smaller equilibrium volume, with no significant change in diastolic curve profile, would substantiate previous invasive studies performed in a smaller study reporting a simple reduction in ventricular volume post-CRT, with no change in stiffness [26].

In our study, the reverse remodelling effect of CRT was associated with no statistically significant change in filling pressures, but with an improvement in the filling profile that seems to have prognostic relevance. Survival analysis, in fact, proves that patients with reduction in ΔE/DT greater than 18 % post-CRT exhibit better survival based on the composite clinical endpoint of worsening heart failure or cardiac death (Fig. 3). The combination of improved filling and reverse remodelling could possibly be related to a reduction in convective deceleration due to decrement in turbulent forces associated with cardiomegaly [27, 28]. Unfortunately, there was no difference in Vp (available before and after CRT in 53 patients only) between the two groups created by dividing the population according to ΔEDV_20_ <>5 %, although overall Vp increased in both groups after CRT (+4.9 ± 12.8 vs. + 5.8 ± 17.9 cm/s, interaction *p* = ns). There was, instead, a significant interaction (*p* = 0.002) between time changes in Vp and CRT when ΔE/DT <>18 % was used as a grouping criterion (−2.4 ± 15.2 vs. +10.3 ± 11.2 cm/s). Such findings are compatible with ventricular overfilling in patients doing badly after CRT and do not conflict with the hypothesis of a modest increment in apical gradient in those improving after CRT [29, 30].

## Limitations

Operator-dependency of all the measured echocardiographic measurements is an unavoidable limitation. In practical terms, this limits the reliability of the data acquisition process. In this regard NMR data would have been more reliable, but difficult to obtain in this patients’ population.

The experimental data were partly biased by not calculating SR_ivr_ in those patients with an inadequate apical 4-chamber and/or 2-chamber view. Thirdly, DF was not evaluated via invasive measurements of pressure and volume but exclusively inferred from echocardiographic parameters. It should be considered, however, that the estimate of EDPVR from a single beat has demonstrated a good correlation with EDPVR measured with invasive techniques, as reported by Klotz et al. [6, 7]. Thus, EDV_10–20–30_ can be considered as a reliable parameter for describing the ventricular passive properties and can be used to describe the process of reverse remodeling after CRT as long as the original input is correct. Given the limitations of E/E′-based measurements of filling pressures in patients with conduction abnormalities or undergoing pacing [18], we used K_lv_/EDV as another parameter, beyond single-beat EDPVR, in order to confirm the changes in ventricular characteristics post-CRT.

Another limitation to be taken into consideration is the relatively small sample size and short follow-up time at which echocardiographic data were obtained. Expansion of the patients’ population and another echocardiographic assessment, after 6 or 12 months, would have allowed verification of the positive trend in the filling profile and echocardiographic parameters of relaxation. In this regard it has to be underlined that the use of the E–E′ time interval, instead of the indexes here considered, could have been better descriptor of the relaxation behavior pre/post-CRT [31]. Unfortunately, in our data, the nonsimultaneous acquisition of the 2 peaks precluded any reliable time-interval computation [32].

Also the time of first evaluation and diagnosis of heart failure and patient’s comorbidity (Table 2) may be of importance in the recurrence of hospitalization and death. So we decided to include diabetes and/or renal failure (defined as creatinine clearance <60 ml/min) as categorical covariates in the Cox analysis. There were no substantial changes in the final results, with age (*p* = 0.017), ischemic etiology (*p* = 0.007) and worsening in LV filling characteristics (ΔE/DT% *p* = 0.033) predicting HF exacerbations or death during follow-up. The statistical weight of ΔEDV_20_% decreased slightly (*p* = 0.068), There was no contribution at all from ΔESV% (*p* = 0.658), QRS duration (*p* = 0.533) and presence of diabetes and/or chronic renal insufficiency (*p* = 0.138).

## Conclusions

Our study demonstrates that CRT improves systolic function and ventricular dyssynchrony and in particular it positively affects DF through a reverse remodeling process due to a left shifting towards a smaller equilibrium volume, with no change in the slope of EDPVR. Such evolution is associated with a favorable impact on the filling profile, although direct indexes of improved active relaxation are not significantly affected.

Finally, short-term changes in DF, together with ventricular volume shrinkage, rather than improvement in systolic function, predict clinical prognosis long-term post-CRT. Such a combination (improved filling coupled with reverse remodelling), rather than amelioration in systolic pump performance, seems to modulate long-term follow-up in these patients.
